# Bryostatin enhances CD20 CAR-T therapy efficacy against B-cell lymphoma by overcoming trogocytosis-mediated antigen loss

**DOI:** 10.3389/fimmu.2025.1748634

**Published:** 2026-01-22

**Authors:** Xiaofeng Wang, Keran Sun, Zhengzheng Zhang, Ling Zhang, Ling Cui, Shuxia Song, Lin Wei

**Affiliations:** 1 Department of Immunology, School of Basic Medical Sciences, Hebei Medical University, Shijiazhuang, Hebei, China; 2 Key Laboratory of Immune Mechanism and Intervention for Major Diseases of Hebei, Shijiazhuang, Hebei, China

**Keywords:** antigen loss, B-cell lymphoma, bryostatin, CD20 CAR-T, trogocytosis, MEK/ERK pathway

## Abstract

**Introduction:**

Trogocytosis, an active membrane transfer process, impairs the therapeutic efficacy of CAR-T cells by inducing antigen loss from tumor cells. This study investigated whether Bryostatin, a PKC modulator derived from marine organisms, could enhance CD20 CAR-T cell activity by up-regulating the CD20 antigen on tumor cells and promoting T cell activation, differentiation and function.

**Methods:**

CD20 antigen expression and trogocytosis-mediated membrane transfer were assessed by flow cytometry and immunofluorescence following co‑culture of CD20 CAR‑T cells with Raji or BALL‑1 cells. Trogocytosis‑positive (Trog⁺) CAR‑T cell cytotoxicity and fratricide by fresh CAR‑T cells were evaluated by ELISA. Proteomic profiling compared metabolic features of Trog⁺ and Trog⁻ CAR‑T cells. Using flow‑sorted BALL‑1 subsets with differential CD20 expression (CD20^low^, CD20^mid^, CD20^hi^), we examined how antigen density affects CAR‑T persistence and killing. Finally, Bryostatin‑mediated CD20 upregulation and its therapeutic impact on CAR‑T efficacy were tested *in vitro* and in a murine subcutaneous lymphoma model.

**Results:**

Upon contact with Raji or BALL-1 cells, CD20 CAR‑T cells underwent trogocytosis, leading to marked loss of tumor‑cell CD20 and impaired cytotoxicity of trogocytosis‑positive (Trog⁺) CAR‑T cells, which also became susceptible to fratricide. CD20 antigen density positively correlated with CAR‑T killing efficacy. Proteomic analysis revealed that Trog⁺ CAR‑T cells exhibited enriched activity in ribosome biogenesis, mRNA surveillance, and RNA catalysis, suggesting elevated protein synthesis alongside exhaustion features. Key MEK/ERK‑related transcription factors (c‑JUN, TCF7) linked to T‑cell activation were downregulated in Trog⁺ cells. In both *in vitro* and mouse lymphoma models, Bryostatin potentiated CD20 CAR‑T‑mediated tumor suppression. Mechanistically, bryostatin upregulated CD20 expression in tumor cells via the MEK/ERK pathway and enhanced c‑JUN/TCF7 levels in CAR‑T cells, promoting their tumor infiltration.

**Conclusion:**

Bryostatin enhances CD20 CAR‑T efficacy by counteracting trogocytosis‑driven antigen loss and upregulating CD20 expression, providing a promising strategy to overcome antigen escape in lymphoma therapy.

## 1 Introduction

ALL is the second most common acute leukemia in adults, with over 6,500 new U.S. cases each year and approximately 75% of cases derived from B-cell precursors. Despite the use of intensive chemotherapy and allogeneic stem cell transplantation, long-term remission is achieved in only about 40% of patients (1), leaving a substantial portion with limited durable benefit. Consequently, the management of relapsed or refractory (R/R) B-ALL represents a significant unmet medical need. CAR-T (Chimeric antigen receptor T) cell therapy has emerged as a pivotal and timely innovation in this setting, offering a promising pathway for patients with otherwise limited options. CD19, CD22, and CD20 are lineage-specific surface antigens broadly expressed on B cells and B-cell lymphomas, making them prime targets for CAR-T therapy (1). Among these, CD19 CAR-T cells have shown impressive remission rates from 70 to 90% in relapsed/refractory B-acute lymphoblastic leukemia (B-ALL) patients in children and adults (2–4). However, 30-70% of patients eventually relapse one year after receiving CD19 CAR-T (5, 6). This relapse is largely driven by the loss of target antigen expression on tumor cells, a major mechanism of therapeutic resistance. While genetic alterations (e.g., alternative splicing of CD19 mRNA) can lead to permanent antigen loss (7), a dynamic and reversible process called “trogocytosis” has recently been recognized as a critical contributor. Trogocytosis is an active process whereby immune cells, including CAR-T cells, strip antigens (such as CD19 or CD20) from the surface of tumor cells and incorporate them onto their own membrane (8, 9). This transfer has a dual detrimental effect: it reduces antigen density on tumor cells, impairing CAR recognition and cytotoxicity, and it can render the antigen-acquiring CAR-T cells targets for destruction by other CAR-T cells (fratricide) (10, 11). Thus, trogocytosis represents a significant barrier to durable CAR-T efficacy.

Given the significant relapse rates attributed to antigen escape, including trogocytosis, there is a pressing need to develop strategies that target alternative or multiple B-cell antigens, or to counteract antigen loss mechanisms themselves. Among the potential targets, CD20 represents a compelling candidate due to its distinct biological role and expression profile. CD20, a membrane-bound protein that contains four trans-membrane domains and a large extra-cellular loop, is involved in B-cell activation, differentiation and cell cycle progression (12, 13). More importantly, CD20 is highly expressed in over 95% of B-cell lymphomas, such as diffuse large B-cell lymphoma, follicular lymphoma, and classical Hodgkin’s lymphoma (13, 14). To overcome antigen loss variation, multi-targeted CAR-T strategies, including CD19/CD20 and CD19/CD22 bispecific or tandem CARs, and CD19/BCMA (B-Cell Maturation Antigen) dual-targeting CAR T-cell therapy (15–20), have been developed. Despite its promise as a CAR-T target, heterogeneity in CD20 expression, particularly low or absent expression in some cases of diffuse large B-cell lymphoma, is associated with poor prognosis (21). Furthermore, approximately 40-50% of patients developed resistance or relapse after treatment with anti-CD20 monoclonal antibody therapy due to loss of the CD20 target antigen (22, 23). This loss can occur through mechanisms including decreased transcriptional or translational expression and, notably, through trogocytosis (“CD20 shaving”) (24).

Although low-affinity CAR designs have been suggested to reduce trogocytosis (25), these approaches may not be ideal for patients with tumor cells that have inherently low antigen density. Similarly, a report indicated that CTL with low affinity stripped peptide-MHC complex from target cells and transferred it to CTL through trogocytosis, yet failed to trigger the killing of tumor cells (26). Therefore, complementary strategies such as pharmacological up-regulation of antigen expression represent an attractive alternative to mitigate trogocytosis and enhance CAR-T efficacy.

Bryostatin-1, a marine-derived macrolide, acts as a protein kinase C (PKC) modulator with a distinct structural profile compared to typical PKC agonists such as diacylglycerol (DAG) and phorbol esters. Due to its unique binding mode, Bryostatin-1 triggers divergent downstream signaling effects. For instance, while phorbol esters often promote tumor cell proliferation, Bryostatin-1 either inhibits or does not affect tumor growth (27). Its favorable safety profile has been demonstrated in clinical trials across cancer, HIV, and Alzheimer’s disease (28–30). Prior researches have demonstrated that Bryostatin had the ability to increase BL22 (an engineered antibody directed against CD22) cytotoxicity and promote the cytotoxic activity of CD22 CAR-T by increasing the expression of CD22 in Raji cells (31, 32). Whether Bryostatin could enhance CD20 expression and improve CD20 CAR-T efficacy, particularly under conditions of antigen loss, has not yet been investigated.

In brief, we assessed the effects of Bryostatin on CAR-T cell cytotoxicity and persistence both *in vitro* and *in vivo*, revealing a novel mechanism by which Bryostatin enhances therapy efficacy through the reversal of trogocytosis-mediated antigen loss.

## 2 Materials and methods

### 2.1 Generation of CD19 CAR-T and CD20 CAR-T cells

CD20 chimeric antigen receptor (CAR) used in this study consists of a nanobody-derived single variable domain on a heavy chain (VHH) sequence targeting CD20, a CD28 hinge and transmembrane domain, a 4-1BB (CD137), CD3-ζ chain signaling domains and T2A autocleavage sequences, and an endodomain-deleted EGFR (tEGFR). The CD19-CAR is composed by anti-CD19 scFv (FMC63), and the intracellular signal domain is the same as that of CD20 CAR. The lentivirus package was performed as described previously (33).

Human PBMCs were isolated from healthy donors at Hebei Provincial Hospital of Traditional Chinese Medicine in Shijiazhuang, China. CD3^+^T cells were sorted using anti-CD3 magnetic beads (Becton, Dickinson and Company, BD), and then activated with anti-CD3/CD28 antibodies (BD). Activated T cells were transduced with CD20 or CD19-CAR LV in 24-well plates in the presence of 8 µg/mL protamine sulfates in TexMACS medium(Miltenyi)supplemented with IL-2 (200 IU/mL, Miltenyi Biotec). Two days later, CAR T cells were stained for tEGFR (Abcam) and the percentage of CAR positive T cells was determined by flow cytometry. On day 4, the cultures were harvested and transferred to a 60 mm culture dish and expanded in it for another 10 to 12 days.

For the CAR-T cell proliferation experiment, the CAR-T cells on day 6 were cultured in 24-well plates(1×10^6^/well), and some cells were taken out for counting every 24 h.

### 2.2 Cell lines and culture conditions

CD20^+^ B-cell leukemia cell lines (Raji, BALL-1) and CD20^-^ chronic myeloid leukemia cells (K562) were cultured in RPMI-1640 (Hyclone) supplemented with 10% fetal bovine serum (FBS, Hyclone) and 1% penicillin/streptomycin.

BALL-1 cells were labeled with antibody of CD20 (APC-labeled ab269316), CD20^high^BALL-1, CD20^mid^BALL-1 and CD20^low^BALL-1 subsets were sorted using fluorescence-activated cell sorting (FACS) (BD FACSAria Fusion, Becton Dickinson), based on CD20 surface expression.

### 2.3 *In Vitro* trogocytosis assay

CAR-T cells and target cells were co-cultured in TexMACS at 37°C/5% CO_2_ for 3 or 12 h at the effector: target ratio of 1:1. Cells were stained with the antibodies CD3 (PE-labeled, BD 557851), CD19 (FITC-labeled, ab1167), CD20 (APC-labeled ab269316), and then the expression levels of CD19 and CD20 were quantified on CAR-T cells or tumor cells, respectively by Flow Cytometry.

### 2.4 Trogocytosis-positive CAR-T cells sorting

CD20 CAR T cells were labeled with CTV(Thermo Fisher Scientific)according to manufacturer’s protocol and co-cultured with BALL-1 cells at a 1:1 effector-to-target ratio for 3 h in TexMACS medium. Then, CAR T cells were stained for tEGFR and CD20 as described above and subsequently sorted by FACS to isolate CTV^+^EGFRt^+^CD20^+^(trog^+^) and CTV^+^EGFRt^+^CD20^-^(trog^-^).

### 2.5 Cytolysis assays

#### 2.5.1 Lactate dehydrogenase release method

The cytolytic activity of CAR-T cells was measured with the LDH-releasing assay using the Cytotoxicity Detection Kit (Takara-Bio). The assay was performed as described previously (34). Briefly, the CAR-T and tumor cells were added at ratios of 5:1, 2.5:1 or 1:1 to the U-shaped 96-well cell culture plate, then, TexMACS medium was added to make the total volume of each well 200 μL. The cell culture plates were centrifuged at 1000 rpm for 5 min at RT and then cultured at 37°C for 16 h. Cells lysed by adding Triton X-100 (Merck, Darmstadt, Germany) to a final concentration of 1% to control wells served as maximum controls. Absorbance was assessed at a wavelength of 490 nm with a reference wavelength of 690 nm on a Microplate Reader (Biotek, Winooski). Lysis (%) was calculated according to the formula: killing efficiency % = (OD value of co-culture well-OD value of natural release well of effector cells-OD value of natural release well of target cells /(OD value of maximum release well of target cells-OD value of natural release well of effector cells).

The amount of IL-2, IFN-γ and GZMB released by effector cells in the presence of target cells was detected by ELISA(Lianke-Bio. EK102,EK180,EK158). Each group was added with 1×10^6^ effector cells and 1×10^6^ tumor cells (1×10^6^ trog^+^/trog^+^ CAR-T cells or PBMCs for indicated experiments) in a 96-well plate, and co-cultured for 24 h. The culture system was TexMACS medium, and the final volume was 200 μL.

#### 2.5.2 Flow cytometry

2×10^5^ target cells (Raji, BALL-1, or K562) were co-cultured with normal T cells or CD20 CAR-T cells at an effector to target ratio of 5:1 in 24-well plates and incubated in TexMACS medium with or without 1ng/ml Bryostatin for 24 h, then, the cells were stained with the antibody of CD107a (PE-labeled, eBioH4A3, eBioscience™) to detect by FCM.

### 2.6 Confocal microscopy

Raji cells expressing a CD20–GFP fusion and CAR-T cells were seeded at 1:1 ratio onto poly-L-lysine-coated glass surface chamber slide. Cells were then co-cultured at 37 °C for 4 h. The slides were fixed with 4% fresh para formaldehyde for 10 min at room temperature, permeabilized with 0.1% TritonX-100 in PBS for 20 min, and then blocked with 5% bovine serum albumin (BSA) at room temperature for 1 h. Subsequently, the cells were stained with iFluorTM 647-Phalloidin (10μg/ml, 200μL/well. CST) for 2 h at room temperature in the dark. All slides were counterstained with DAPI for 5 min. Confocal microscopy (Carl Zeiss, LSM 710) was employed to observe the distribution of F-actin and immune synapse formation.

### 2.7 Bryostatin treatment and CD20 expression analysis *in Vitro*

Raji or BALL-1 cells (3×10^5^) or PBMCs (3×10^5^) from patients with acute B lymphoblastic leukemia were cultured in 12 well plates with or without 0.25 up to 10 ng/ml Bryostatin (MCE, HY-105231) for 12h or 24h, respectively. CD20 mRNA levels were assessed by qRT-PCR, and CD20 protein expression was measured by flow cytometry. Cell viability was determined using MTS assays Kit (Abcam, ab197010) according to the instructions.

### 2.8 Western blot

2×10^5^ Raji cells, which were treated with 0, 1 or 10 ng/ml Bryostatin for 24h. Cell lysates were separated by 10% SDS-PAGE and transferred to nitrocellulose membranes (GE Healthcare, USA). After being blocked with 5% milk in TBS for 1.5 h at room temperature, the membrane was incubated with specific primary antibodies (anti-ERK1/2, ab184699, anti-ERK1(phosphor T202+Y204), ab278538) at 4°C for 12 h, followed by secondary antibodies at room temperature for 1h. Protein visualization was conducted by ECL chromogenic substrate, quantification of protein expression was carried out using ImageJ software.

### 2.9 Quantitative real-time PCR

Total RNA was extracted using Trizol. Quantitative real-time PCR was performed using SYBR Green PCR kits (Takara, Japan). The following primers sequences were used: CD20: up: 5’AACTCAGCAGTAGGCCTTGC3’, down: 5’GCAGTCTTACCTTGTGTCATGC3’(Sangon Biotech (Shanghai) Co., Ltd.); β-actin: up:5’AAGGTGACAGCAGTCGGTT3’, down: 5’-TGTGTGGACTTGGGAGAGG-3’ (Sangon Biotech (Shanghai) Co., Ltd.).

### 2.10 LC-MS/MS analyses

Proteins from fresh, trog^−^, and trog^+^ CAR-T cells were quantified and digested with trypsin. The digest peptides were desalted, and concentrated to 40 µl of 0.1% (v/v) formic acid. LC-MS/MS analyses were conducted using a Q Exactive mass spectrometer (Thermo Scientific) coupled with an Easy nLC system (Proxeon Biosystems). The total LC separation time was 65 minutes. The peptides were loaded onto a reverse phase trap column (Thermo Scientific Acclaim PepMap100, 100 μm× 2 cm, nanoViper C18) connected to the C18-reversed phase analytical column (Thermo Scientific Easy Column, 10 cm long, 75 μm inner diameter, 3μm resin) in buffer A (0.1% Formic acid) and separated with a linear gradient of buffer B (84% acetonitrile and 0.1% Formic acid) at a flow rate of 300 nl/min controlled by IntelliFlow technology. The mass spectrometer was operated in positive ion mode. MS data was acquired using a data-dependent top10 method dynamically choosing the most abundant precursor ions from the survey scan (300–1800 m/z) for HCD fragmentation. Automatic gain control (AGC) target was set to 3e6, and maximum inject time to 10 ms. Dynamic exclusion duration was 40.0 s. Survey scans were acquired at a resolution of 70,000 at m/z 200 and resolution for HCD spectra was set to 17,500 at m/z 200, and isolation width was 2 m/z. Normalized collision energy was 30 eV and the underfill ratio, which specifies the minimum percentage of the target value likely to be reached at maximum fill time, was defined as 0.1%. The instrument was run with peptide recognition mode enabled.

Unique protein signatures were analyzed using KEGG and Gene Ontology (GO) enrichment via the DAVID platform.

### 2.11 *In Vivo* tumor models

A total of 20 6-wk-old male nude mice (BALB/c) weighing 20.08 ± 1.34g was purchased from Beijing Speiford Biotechnology Co., LTD). The Raji cells were suspend with pre-cooled PBS (1×10^7^ Raji cells in 75 µL for each mouse) and mixed with an equal volume of Matrigel (Corning. Matrigel is stored in a -80°C freezer and thawed on ice), resulting in a mixture of 75µl of cells and 75 µLof Matrigel for mouse. The Raji cells were inoculated subcutaneously into the right armpit of the mice 48h after receiving intraperitoneal cyclophosphamide (100mg/mouse). Then, the mice were randomized into a Bryostatn (40 μg/kg) group, a CD20 CAR-T-cell group, a Bryostatn combined with CD20 CAR-T cells group, and a control (PBS) group. There were 5 mice in each group. CAR-T cells (5×10^6^/mouse/per injection) were administered via the tail vein on days 7 and 14 after tumor inoculation, respectively. Bryostatin was injected intraperitoneally the day after the CAR-T injection. All mice were sacrificed on the 43rd day of tumor bearing.

After the mice were injected with CAR-T cells, the diameter of the tumor mass was measured every three days, the volume of the tumor mass was calculated, and the tumor growth curve was plotted. On the 43rd day after tumor loading, the mice were sacrificed under CO_2_ anesthesia, the tumor tissues were stripped, photographed and weighed.

To observe the retention of CAR-T cells in the blood of mice, peripheral blood was collected from the mice on the 1st, 3rd, 7th, and 14th days after receiving CAR-T cell therapy. The blood was collected through the tail vein. First, anti-mouse CD16/CD32 blocking antibodies were added. After twenty minutes, anti-mouse CD3 antibodies were added again. The frequency of CD3-positive cells was then detected by flow cytometry.

### 2.12 Immunofluorescence

Tumor tissues were fixed with 4% paraformaldehyde for 48 h, rinsed with PBS, dehydrated with gradient ethanol, transparent with xylene, and embedded in paraffin to prepare sections (4 μm). Subsequently, the sections were incubated with FITC-labeled anti-CD3 antibody and CD20 Alexa Fluor 350 antibody (1:200, BioLegend) overnight at 4°C. After washing with PBS, the cell nuclei were counterstained with DAPI (BioLegend) and the sections were sealed with neutral gel and were monitored using a fluorescence microscope (Zeiss, LSM710).

### 2.13 Statistical analysis

The data processing software involved in this study includes FlowJo V10 and ImageJ. All data were statistically analyzed using GraphPad Prism 9.5 statistical software. For measurement data with normal distribution and uniform variance, the two-sample mean comparison was conducted using the two-independent sample t-test, and ordinary one-way ANOVA was used for the multi-sample mean comparison. The rank sum test was used for measurement data with unequal variances. Measurement data were expressed as mean ± SD. Differences with P < 0.05 were considered statistically significant.

## 3 Results

### 3.1 Trogocytosis is initiated upon the interaction between CAR-T cells and tumor cells

**Figure 1:**
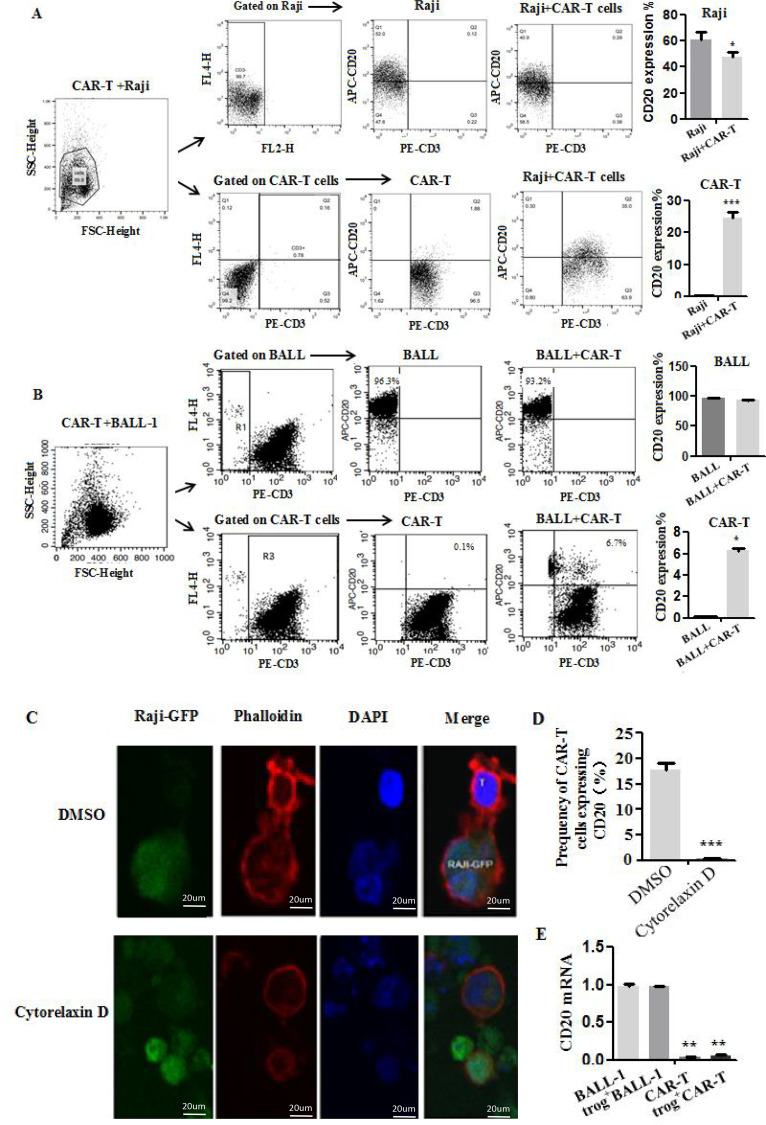
Trogocytosis is initiated upon the interaction between CAR-T cells and tumor cells. **(A, B)** Percentage of CD20 on Raji or BALL-1 and CAR T cells after co-culture of CD20 CAR-T and tumor cells detected by flow cytometry. Data were collected at 3 h after co-culture gated on live singlet cells (n=3 independent samples). *P<0.05,**P<0.01, ***P<0.001. **(C)**. Representative confocal microscopy images of Raji-GFP cell–CAR-T cell conjugates. Data are representative of two experiments. **(D)** Cytochalasin C reduces CD20 expression on CAR-T cells by inhibiting trogocytosis occurrence. **(E)** The level of CD20 mRNA on trog^+^ CAR-T cells. As with fresh CAR-T cells, mRNA expression were not detected in trog^+^ CAR-T cells.

To investigate whether trogocytosis mediates upon the interaction between CAR-T cells and tumor cells, we firstly generated CD19 CAR-T and CD20 CAR-T cells using retroviruses carrying chimeric receptors targeting CD19 or CD20 antigens (**Supplementary Figure S1A**), which were provided by Hebei Senlang Biotechnology Co., Ltd. Higher expression of CARs and cytotoxic activity of CAR-T cells were observed respectively by flow cytometry or LDH (**Supplementary Figures S1B, C**). Then, CD20 CAR-T cells with Raji or BALL-1 cells were co-cultured at a 1:1 effector-to-target (E:T) ratio for 3 h. It was observed that the expression of CD20 in Raji cells was significantly decreased, while approximately 24% of CD20 CAR-T cells were detected with CD20 antigen (**Figure 1A**). Although the CD20 antigen of BALL-1 cells remained relatively stable, a notable increase in CD20^+^ CAR-T cells were still observed after co-culture (**Figure 1B**). Furthermore, prolonged co-culture (from 3 to12 h) intensified the reduction of CD20 on Raji cells in a time-dependent manner, suggesting a progressive antigen loss reliant on CAR-T interaction (**Supplementary Figure S2**). In contrast, normal T (NT) cells lacking CAR expression failed to induce any detectable antigen loss after targeting the Raji cells, confirming that trogocytosis is a CAR-specific event. In addition, we also confirmed similar findings with CD19 CAR-T cells (**Supplementary Figure S3**), further supporting that trogocytosis occurs upon the interaction between CAR-T cells and tumor cells.

The process of trogocytosis involves the transfer of membrane proteins between two contacting cells. Thus, the formation of immunological synapses and actin dynamics were visualized by the confocal microscopy on Raji-GFP cells co-cultured with CD20 CAR-T cells. After phalloidin staining, the formation of synapses between the two cells was clearly observed (**Figure 1C**). Moreover, treatment with cytochalasin C, an inhibitor of actin polymerization, could also significantly inhibit trogocytosis occurrence between them, followed by reduction in transferring of CD20 to CAR-T cells (**Figure 1D**), suggesting that actin remodeling is essential for trogocytosis. Therefore, the initiation of trogocytosis occurs through the interaction between CAR-T cells and tumor cells, which may be facilitated by actin-mediated synapse formation.

### 3.2 Trogocytosis impairs CD20 CAR-T cytotoxicity associated with hyperactive transcriptional and metabolic profiles

Next, we examined the influence of trogocytosis on the cytotoxic function of CD20 CAR-T cells. Following a 3-hour co-culture with BALL-1 cells, CD20^+^CAR-T cells that had acquired CD20 via trogocytosis (Trog^+^) were sorted using flow cytometry. Quantitative PCR confirmed the absence of CD20 mRNA in Trog^+^CAR-T cells (**Figure 1E**), indicating surface acquisition rather than endogenous expression for the transfer of membrane proteins between donor and recipient cells in the presence of trogocytosis.

**Figure 2:**
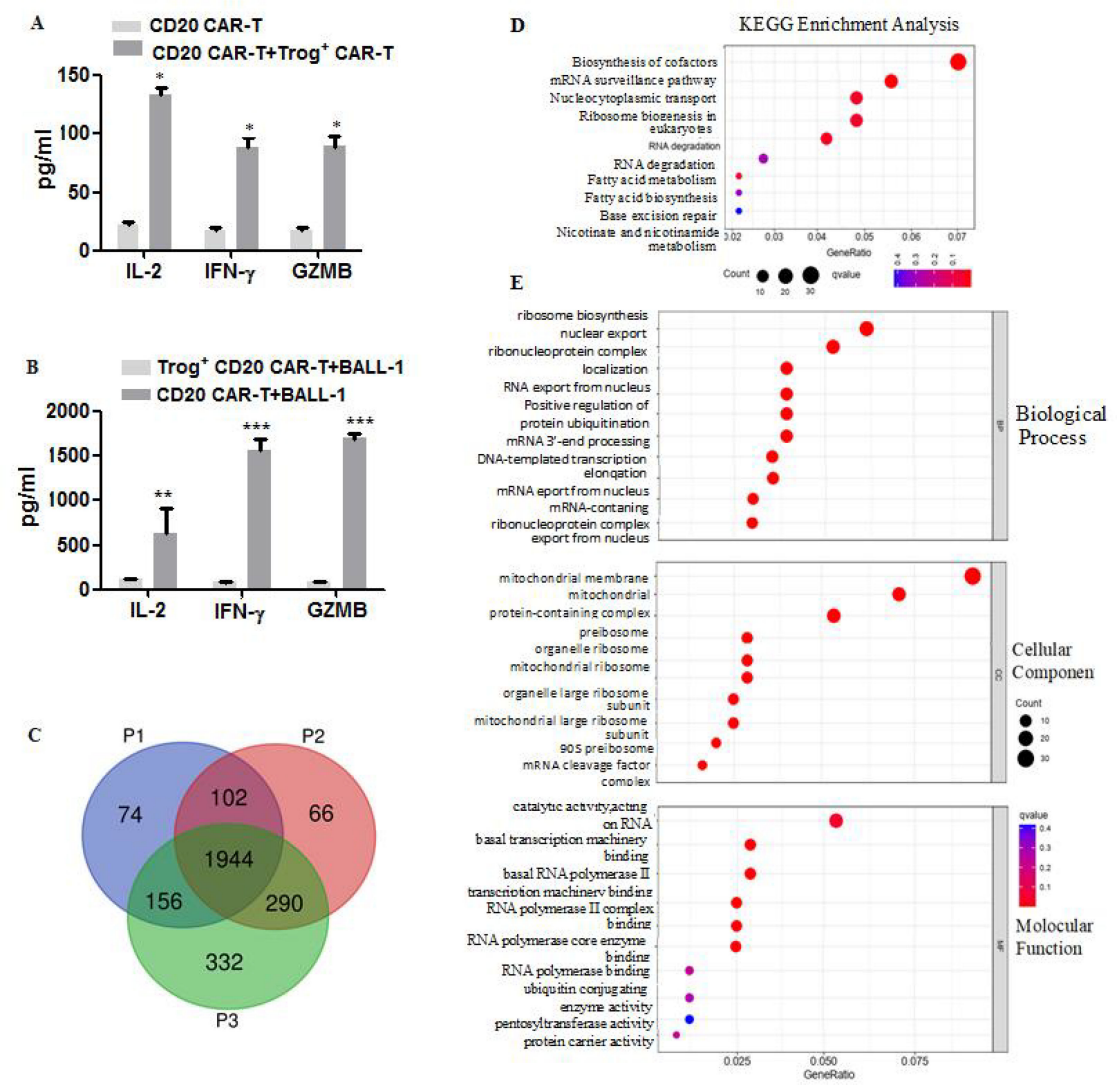
The killing activity of trog^+^ CAR-T cells changed after trogocytosis. **(A)** The levels of IL-2, IFN-γ and GZMB in the supernatants after fresh CD20 CAR-T co-culturing with trog^+^ CAR-T cells detected by ELISA. **(B)** The levels of IL-2, IFN-γ and GZMB in the supernatants following fresh or trog^+^ CD20 CAR-T cells co-culturing with BALL-1 detected by ELISA. *P<0.05, **P<0.01, ***P<0.001 (n=3 independent samples). Protein identification of trogocytotic CAR-T cells was performed based on LC-MS. **(C)** Venn figure. **(D)** KEGG enrichment analysis. **(E)** GO enrichment analysis.

To evaluate the risk of fratricide targeting Trgo^+^ cells, Trog^+^ CAR-T cells were co-cultured with fresh CD20 CAR-T cells at a 1:10 ratio. Analysis after 16 h revealed elevated supernatant levels of IFN-γ, IL-2, and granzyme B, indicating that the fresh effector CAR-T cells were activated by the CD20 antigen present on the Trog^+^ targets (**Figure 2A**). This demonstrates that Trog^+^ CAR-T cells can be misidentified as tumor cells, making them susceptible to fratricide. Furthermore, when Trog^+^ CAR-T cells were co-cultured with BALL-1 tumor cells, their production of these same cytotoxic molecules was significantly reduced compared to fresh CAR-T cells (**Figure 2B**). The results suggest that the cytotoxic activity of Trog^+^ CAR-T cells is severely impaired, resulting in functional exhaustion. Collectively, these findings indicate that CAR-T cells engaged in trogocytosis display the reduced tumor-killing efficacy and are susceptible to fratricide among newly generated CD20 CAR T cells, thereby restricting therapeutic durability.

To analyze the functional changes of Trog^+^ CAR-T cells, we analyzed their expression profiles through proteomics. The key finding is that Trog^+^ cells uniquely express 332 proteins, far exceeding the quantity of Trog^-^ (66) and primordial cells (74). This indicates that Trog^+^ CAR-T cells have a large amount of new protein synthesis (**Figure 2C**, **Supplementary Data**). KEGG pathway analysis revealed that proteins specifically upregulated in Trog^+^ cells (P3 vs P2) were significantly enriched in RNA-related processes, including mRNA surveillance and RNA degradation, suggesting reduced mRNA synthesis and stability. Conversely, enrichment in nucleocytoplasmic transport, fatty acid metabolism, and ribosome biogenesis indicates active protein synthesis (**Figure 2D**). Furthermore, ribosome biosynthesis and RNA export in GO analysis also indicate vigorous protein synthesis (**Figure 2E**, **Supplementary Figure S4A**). Concurrently, trog^+^ CAR-T cells exhibited downregulation of over 440 proteins, including CD70, MAPKAPK2, CCR7, and JUN (**Supplementary Data**), suggesting impaired T cell activation, reduced cytotoxicity, and a diminished capacity for memory differentiation. Moreover, the GO chord plot revealed that the down-regulated genes in trog^+^ CAR-T cells are primarily associated with impaired cytotoxic function. These include genes involved in responses to various viral infections, such as human immunodeficiency virus 1, human cytomegalovirus, and Kaposi sarcoma-associated herpesvirus, as well as processes related to phagosome function, oxidative phosphorylation, and amino sugar and nucleotide sugar metabolism (**Supplementary Figure S4B**). Despite their ongoing metabolic activation, this pattern indicates that trog^+^ CAR-T cells exhibit impaired intracellular signaling and effector function, which aligns with their observed functional deficit.

Collectively, these findings indicate that Trog^+^ CAR-T cells undergo substantial transcriptional and metabolic reprogramming, which may underlie their functional exhaustion despite exhibiting a phenotypically activated state.

### 3.3 Tumor antigen density determines CAR-T cell activation and cytotoxicity

**Figure 3:**
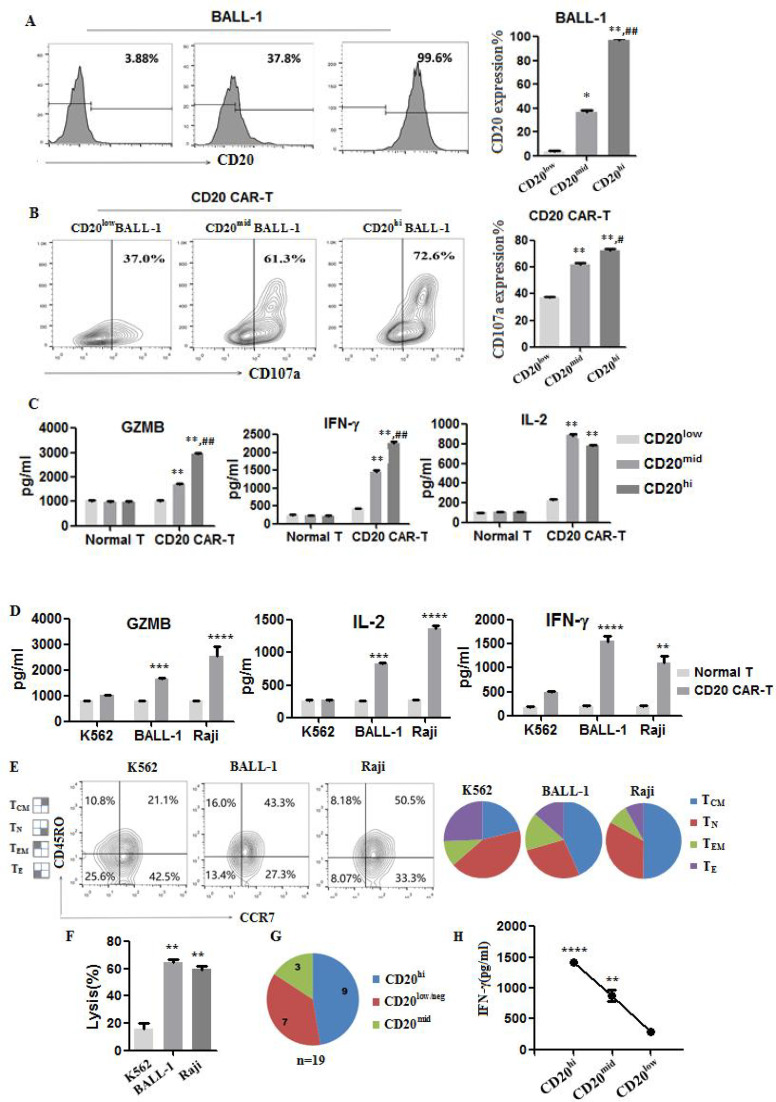
The expression level of CD20 in tumor cells impacts the activation of CD20 CAR-T cells. **(A)** BALL-1 cell line was divided into three subclones, such as CD20^hi^, CD20^mid^ or CD20^neg/low^ according to different expression levels of CD20 antigen. **(B)** CD107a expression on CAR-T cells after 4 h of co-culture with BALL-1 three subclones, respectively. **(C)** IL-2, IFN-γ and GZMB detection after 4 h of CD3^+^CD20 CAR-T or Normal T cells co-culture with BALL-1 three subclones, respectively. Data are representative of three experiments. Compared with CD20neg/low, *P<0.05, **P<0.01 (n=3 independent samples). Compared with CD20^mid^, #P<0.05, ## P<0.01. **(D)** IL-2, IFN-γ and GZMB detection after 4 h of CD20 CAR-T or untransduced T cells co-culture with K562, BALL-1 or Raji cells, respectively. **(E)** Phenotype determined by CD45RO and CCR7 expression in CD3^+^CD20 CAR-T cells. Cells were grouped by flow cytometry according to T-cell phenotype as follows: naïve (TN) CCR7^+^CD45RO^-^, TCM CCR7^+^CD45RO^+^, TEM CCR7^-^CD45RO^+^ and effector (TE) CCR7^-^CD45RO^-^. **(F)** The lysis activity of CAR-T cells. Data are representative of three experiments. **(G)** Surface expression of CD20 in PBMCs from B-ALL leukemia patients was qualified by flow cytometry. According to the different expression levels of CD20, 19 patients were divided into three groups: high-level expression of CD20, moderate expression of CD20 and low-level expression of CD20. **(H)** The higher the expression level of CD20 in PBMCs, the higher the production of IFN-γ after co-culturing with CD20 CAR-T for 4 h. Average of triplicate wells is shown, values differing from untreated controls are indicated, ** indicates P<0.01, ***P<0.001, ****P<0.0001.

The antitumor activity of CAR-T cells is highly dependent on the density of target antigen. When the expression of tumor antigen is below a certain threshold, CARs often cannot exert its antitumor effect (35). To test this, BALL-1 cells were divided into three subsets including CD20 high expression (CD20^hi^), CD20 medium expression (CD20^mid^), and CD20 no expression or weak expression (CD20^neg/low^), based on surface CD20 levels. Flow cytometry confirmed expression rates of 99.8%, 37.8%, and 3.9%, respectively (**Figure 3A**).

Co-culture of CD20 CAR-T cells with each BALL-1 subset revealed a direct correlation between CD20 density and CAR-T activation. Increased levels of antigens resulted in higher CD107a expression (**Figure 3B**), as well as increased secretion of IL-2, IFN-γ, and GZMB (**Figure 3C**). Similarly, when CD20^+^ Raji or BALL-1 but not CD20^-^ K562 was co-cultured with fresh CD20 CAR-T for 16h, the production levels of IL-2, IFN-γ and GZMB were significantly increased (**Figure 3D**). Moreover, nearly half of the CAR-T cells co-incubated with K562 remained phenotypically as naive T cells ((T_N_, CCR7^+^CD45RO^-^), and the number of T_EM_ (CCR7^-^CD45RO^+^) and T_CM_ (CCR7^+^CD45RO^+^) phenotypic cells was the lowest, suggesting that CD20^-^ K562 failed to elicit CD20 CAR-T cells to different into memory T cells (**Figures 3E, F**).

We further examined the expression levels of IFN-γ in the PBMCs of 19 B-ALL patients after co-culture with CD20 CAR-T. The results showed that the higher the expression level of CD20 on PBMCs, the greater the production of IFN-γ (**Figure 3G, H**). Collectively, antigen density is a critical determinant of CAR-T cell efficacy. Insufficient CD20 expression undermines activation and cytotoxic function, suggesting that restoring or augmenting target antigen levels may improve CAR-T therapeutic outcomes.

### 3.4 Bryostatin upregulates CD20 expression via MEK/ERK pathway activation

**Figure 4:**
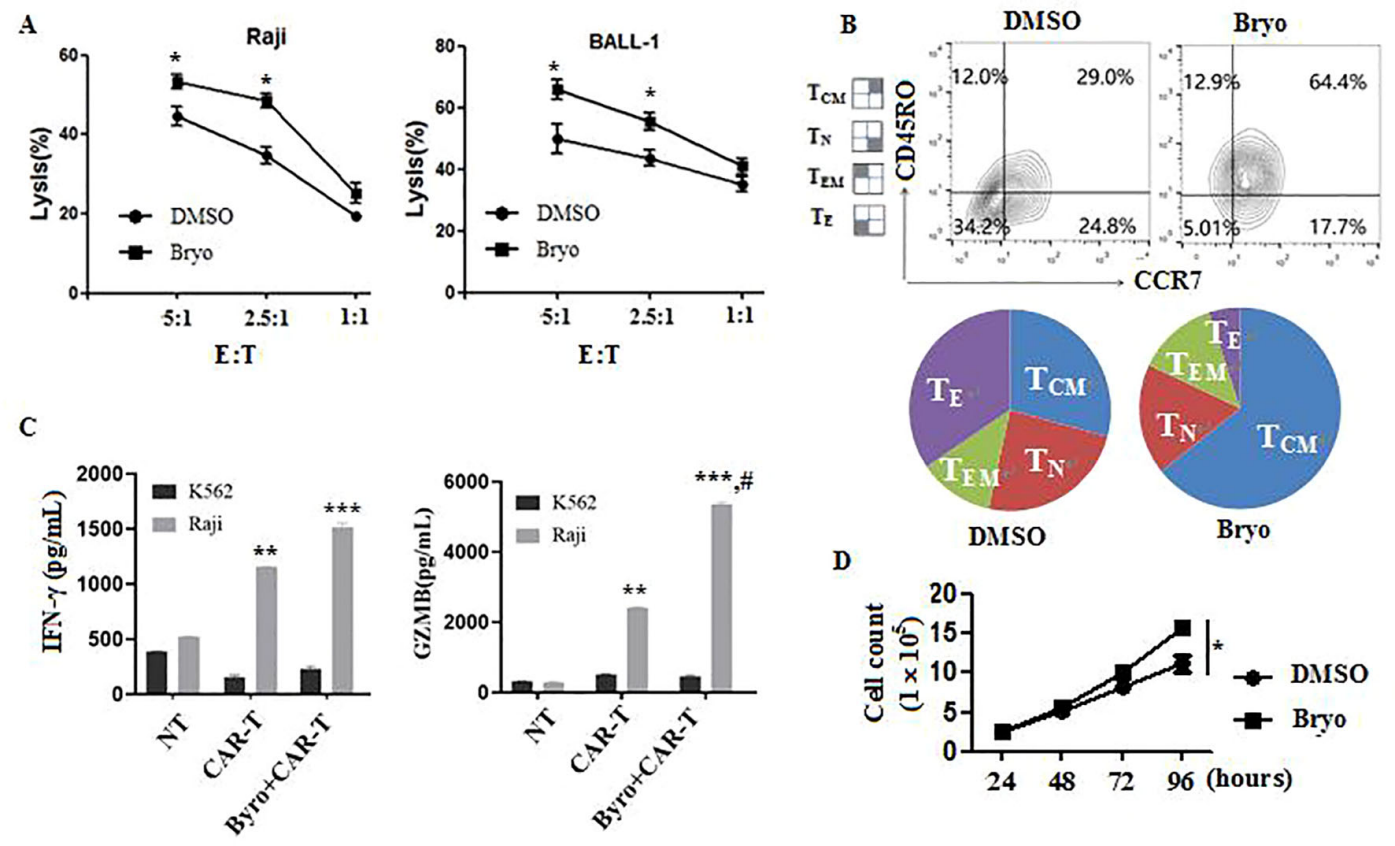
Bryostatin can significantly increase the expression of CD20 in tumor cells and normal B cells. **(A-C)** when Raji, BALL-1 or normal PBMCs were treated with Bryostatin for 24 h, the expression of CD20 was significantly increased. **(D, E)** The expression levels of CD20 mRNA and protein both significantly increased with the prolongation of the treatment time of PBMCs by Byrostatin. **(F)** Byrostatin has no obvious cytotoxicity. **(G)** Bryostatin could induce ERK activation. **(H)** The inhibitor PD98059 of MEK, an upstream signaling molecule of ERK, could significantly down-regulate the expression of CD20 induced by Bryostatin. **(I)** Bryostatin docks with MEK protein molecules. Average of triplicate wells is shown, values differing from untreated controls are indicated, * or # indicates P<0.05, **P<0.01, ***P<0.001 (n=3 independent samples).

Trogocytosis may lower the CD20 antigen on tumor cells, which in turn hinders the ongoing killing ability of CD20 CAR-T cells. To investigate whether Bryostatin can enhance CD20 CAR-T therapy efficacy by increasing CD20 expression, Raji and BALL-1 cells were treated with increasing concentrations of Bryostatin from 0 to 1 ng/mL. Flow cytometry analysis revealed a dose-dependent upregulation of CD20 expression in Raji from 27.5% at baseline to 58.9% at 1 ng/mL, in BALL-1 cells from 64.6% to 72.3%, respectively (**Figures 4A, B**). Similarly, treatment of PBMCs with Bryostatin (1 ng/mL) significantly increased the proportion of CD20-positive cells in comparison between 48.6% at DMSO group and 88.4% at Bryostatin group (**Figure 4C**). Further time-course analysis displayed that CD20 expression was significantly elevated after 12 h and further increased at 24 h post-treatment, compared with baseline at mRNA and protein level, respectively (**Figures 4D, E**). Importantly, Bryostatin did not affect cell viability in either Raji or BALL-1 cells at concentrations up to 10 ng/mL (**Figure 4F**), indicating that its effects on CD20 expression were not attributable to cytotoxicity. These findings suggest that Bryostatin enhances the CD20 expression at the transcriptional and protein levels in a dose- and time-dependent manner, without affecting cell viability.

Consistent with proteomic data, trog^+^ CAR-T cells (P3) shifted from a cytotoxic state to a metabolically hyperactive but dysfunctional profile, enriched in RNA transcription and ribosome biogenesis-processes, aligning with the known downstream activity of MEK/ERK signaling (36, 37), which orchestrated transcriptional and metabolic remodeling in CAR T immune cells (38). To explore how CD20 expression is regulated in Raji cells, we assessed ERK-1/2 phosphorylation after Bryostatin treatment. Western blot revealed dose-dependent increases in phosphorylated ERK (**Figure 4G**). Furthermore, PD98059 inhibited Bryostatin-induced CD20 upregulation concentration-dependently, indicating ERK activation is required for this effect (**Figure 4H**). Molecular docking predicted direct Bryostatin-MEK binding, suggesting a structural mechanism for MEK activation (**Figure 4I**). Together, these findings demonstrate that Bryostatin upregulates CD20 via the MEK/ERK signaling pathway.

### 3.5 Bryostatin enhances the persistent killing activity of CAR-T cells *in vitro*

**Figure 5:**
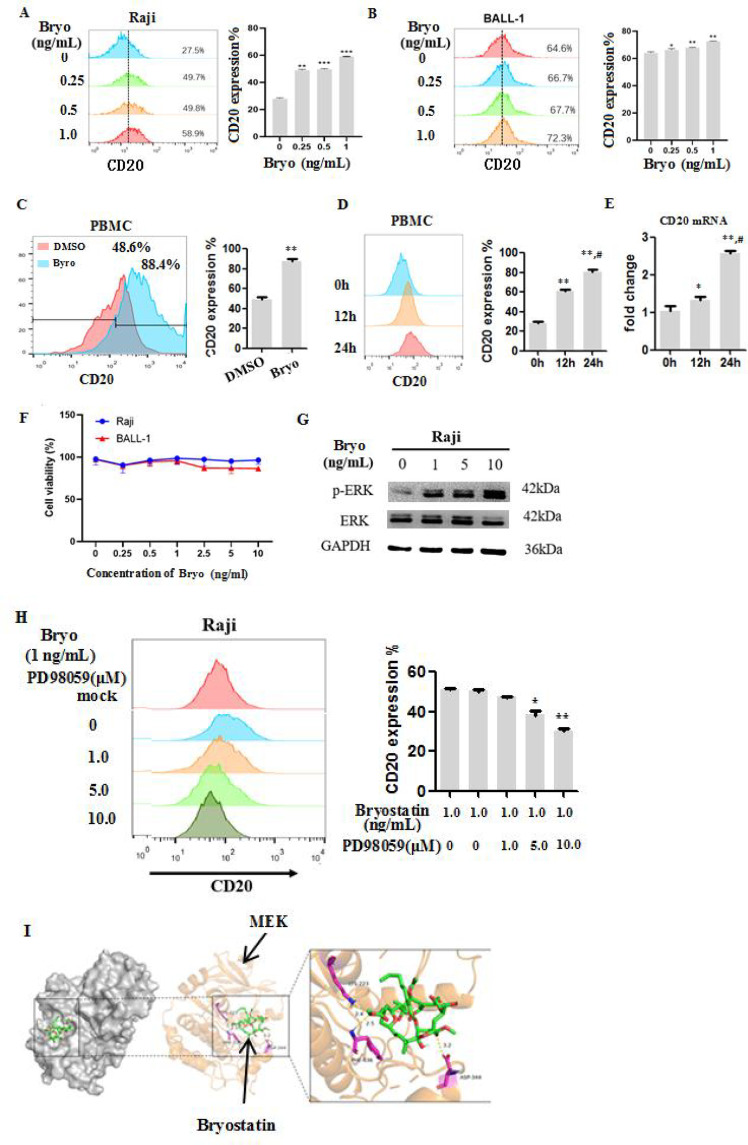
Bryostatin could enhance the killing activity of CD20 CAR-T cells *in vitro*. **(A)** The killing activity of CD20 CAR-T cells on Raji or BALL-1 was detected by LDH release method. Bryostatin significantly enhanced the cytotoxic activity of CAR-T cells. Compared with DMSO, * P<0.05 (n=3 independent samples). **(B)** Flow cytometric quantification of TCM or TN, represents CAR-T cells with long-lasting activity from co-cultures shown in **(A)** Bryostatin significantly enhanced the numbers with TCM and TN phenotype of CAR-T cells. **(C)** Bryostatin significantly increased the production of IFN and GZMB of CAR-T rather than NT after co-cultured with Raji for 4 h respectively. Compared with NT, **P<0.01, ***P<0.001. Compared with CAR-T, # P<0.05 (n=3 independent samples). **(D)** Bryostatin significantly promoted the proliferation of CAR-T cells. Data are representative of three experiments. *P<0.05 (n=3 independent samples).

To determine whether up-regulation of CD20 expression by Bryostatin could enhance CAR-T-mediated tumor cell killing, we performed *in vitro* cytotoxicity assays. The assay was carried out by co-culturing CD20 CAR-T and Raji cells at the ratio of 5:1, 2.5:1 or 1:1 for 24 h with or without 1 ng/ml Bryostatin. As expected, treatment with Bryostatin rendered both Raji and BALL-1 cells more efficiently to CD20 CAR-T-mediated cytolysis (**Figure 5A**). Notably, differentiation phenotype of CD20 CAR-T cells was also observed following co-culture with Raji in presence of Bryostatin (1ng/ml). In comparison to control, central memory T cells (T_CM_) frequencies were significantly higher in CD20 CAR-T cells treated with Bryostatin than those in control group (**Figure 5B**), suggesting improved persistence and long-term activity.

Functionally, cytokine analysis revealed that Bryostatin-treated CAR-T cells secreted higher levels of IFN-γ and GZMB upon co-culture with Raji cells, compared with untreated CAR-T cells (**Figure 5C**). Furthermore, Bryostatin promoted CAR-T cell expansion *in vitro*, resulting in significantly higher cell counts at 96 h compared with controls (**Figure 5D**). All in all, these findings suggest that Bryostatin enhances the persistent killing activity of CAR-T cells *in vitro*, promotes a memory-like phenotype, improves effector cytokine production, and facilitates T-cell expansion.

### 3.6 Bryostatin synergizes with CD20 CAR-T therapy *in Vivo*

**Figure 6:**
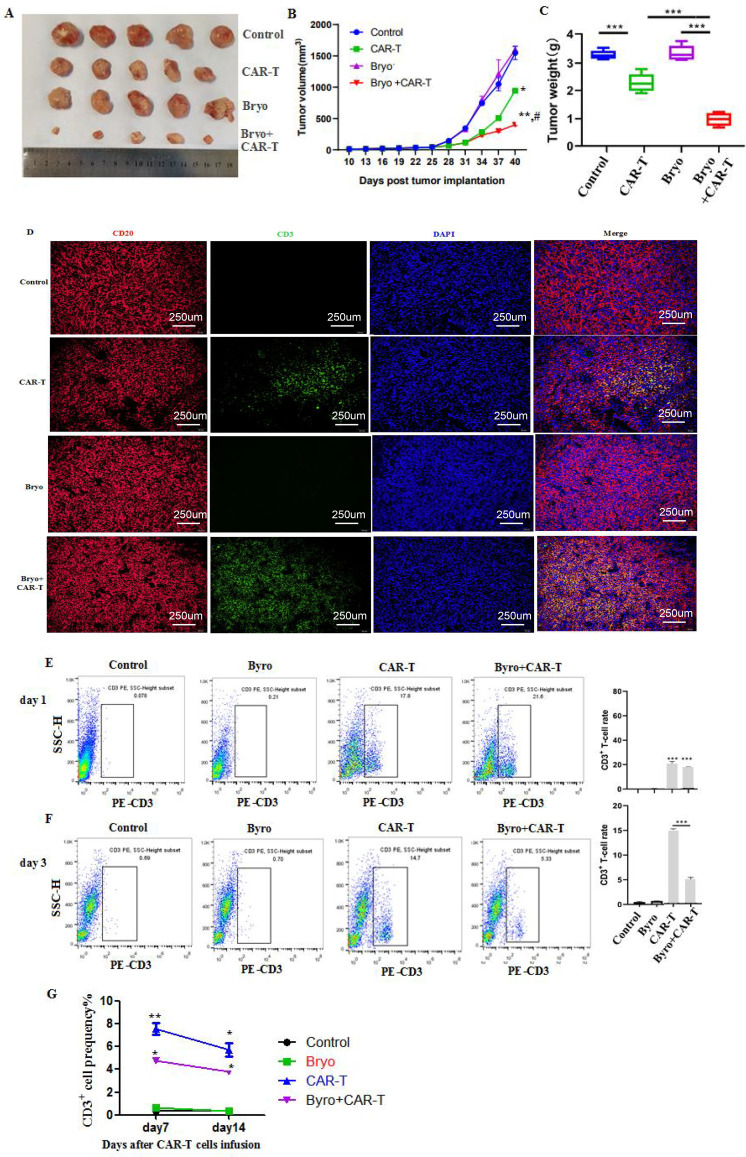
Bryostatin enhanced the growth inhibitory effect of CAR-T on tumors in tumor-bearing mice. **(A–C)** (n=5). Asterisks denote statistical significance as follows: *, **, and *** indicate P<0.05, P<0.01, and P<0.001, respectively; ^#^ indicates P<0.05 versus the CAR-T group. Tumor tissue morphology and size; tumor growth curve; tumor weight (g). **(D)** The expressions of CD3 (green) and CD20 (red) in tumor tissues of tumor-bearing mice were detected by IF. **(E-G)** The frequency of CAR-T cells in peripheral blood of mice. On the 1st, 3rd, 7th and 14th days after CAR-T cell therapy in mice, peripheral blood of mice was collected through the tail vein respectively. After labeling with anti-mouse CD3 antibody, the frequency of CD3-positive cells was detected by flow cytometry. Asterisks denote statistical significance as follows: **, and *** indicate P<0.01, and P<0.001, respectively.

To validate these findings *in vivo*, a subcutaneous xenograft model was established in BALB/c nude mice using Raji cells. Gross examination of excised tumors showed that CD20 CAR-T alone treatment reduced tumor size compared with the control group, whereas the combination of Bryostatin and CD20 CAR-T cells produced the smallest tumors (**Figure 6A**). Tumor growth curves demonstrated that CD20 CAR-T monotherapy significantly delayed tumor progression, while the combined therapy exhibited superior tumor control, compared with CD20 CAR-T alone or Bryostatin alone treatment (**Figure 6B**). At the experimental endpoint, the combination therapy yielded the lowest tumor weight, which was significantly lower than that of either treatment alone (**Figure 6C**). These findings suggest that Bryostatin synergistically enhances the antitumor efficacy of CAR-T cells *in vivo*.

Meanwhile, immunofluorescence staining of tumor tissues (**Figure 6D**) revealed that CD20 CAR-T treatment reduced CD20 expression, indicating antigen loss after killing, while Bryostatin monotherapy enhanced CD20 signals. CD20 (red) staining showed abundant tumor cells in control and Bryostatin-only groups, with diminished intensity in CAR-T treated groups. Conversely, CD3 (green) staining demonstrated significant T-cell infiltration in CAR-T groups, which was further enhanced by Bryostatin combination. These results confirm that Bryostatin upregulates tumor CD20 expression and promotes CAR-T tumor trafficking.

Additionally, to assess the impact of Bryostatin on the *in vivo* persistence of CAR-T cells, we quantified peripheral blood CD3^+^T cells at multiple time points following CD 20 CAR-T infusion. On day 1, CD20 CAR-T treatment significantly increased circulating CD3^+^T cells, compared with the control and Bryostatin monotherapy groups, respectively, and this effect was further enhanced in the combination therapy group (**Figure 6E**). On day 3, CD3^+^ T cell frequency declined in both CD20 CAR-T group (4.80%) and combination group (9.16%), but it remained significantly higher in the combination group (**Figure 6F**). Over time, circulating CD3^+^T cells were found to decrease, but they persisted at relatively higher level of 5.9% ± 0.6% or 3.7% ± 0.4% on the 14th day after CAR-T or combined therapy, respectively (**Figure 6G**). These findings indicate that Bryostatin enhances early expansion and persistence of CAR-T cells *in vivo*, supporting its potential to counteract trogocytosis and improve therapeutic outcomes.

## 4 Discussion

Although CAR-T cell therapy has achieved success, multiple obstacles still limit their effectiveness, including obstacles of the tumor microenvironment, especially in solid tumors, and restrictions on antigen expression to protect oneself from recognition and anti-tumor immune responses (39, 40). Tumor antigen loss not only causes resistance to CAR-T cells and the lack of T cell persistent activity, but is also an important factor for recurrence (41). Trogocytosis, which requires close contact between CAR-T and tumor cells, represents one mechanism driving antigen loss (42). Although CD20 exhibits more stable gene expression than CD19, trogocytosis remains inevitable upon engagement of CD20 CAR-T cells with tumor targets, ultimately compromising treatment efficacy.

It is generally believed that trogocytosis, which occurs when CAR-T cells come into contact with tumors, is an active process of interaction between CAR-T and tumor cells (10, 43). In this study, co-culture of CD20 CAR-T cells with Raji or BALL-1 cells for 3 h led to decreased CD20 expression on tumor cells, while a subset of CAR-T cells became CD20-positive. A similar phenomenon was observed with CD19 CAR-T cells. In contrast, normal T cells, which lack tumor antigen recognition, did not exhibit trogocytosis, indicating that this process is specifically triggered during CAR-T cell-mediated tumor killing. Mechanistically, cytoskeletal remodeling during immune synapse formation between CAR-T and tumor cells is crucial for trogocytosis occurrence (**Figure 1**). Our results further indicate that only activated T cells are likely to undergo trogocytosis. It is crucial to explore how to overcome the influence of trogocytosis on the cytotoxic activity of CAR-T (44).

Trogocytosis induces exhaustion in CAR-T cells, impairing their anti-tumor function while serving as a self-protective mechanism for tumor cells (45–47). In this study, trog^+^ CAR-T cells not only exhibited severely reduced cytotoxicity but also became targets for elimination by fresh CAR-T cells (**Figures 2A, B**). Proteomic analysis revealed significant signal and metabolism rearrangement in trog^+^ CAR-T cells, with numerous proteins expressed at more than twice the level of those in fresh CAR-T cells (**Figures 2D, E**). Recent studies have shown that there are a large number of protein aggregates in exhausted T cells, that is, the collapse of intracellular proteostasis is the disaster of T cell exhaustion (48). Therefore, although protein synthesis in byro^+^CAR-T cells is active, protein folding has become impaired. Conversely, more than 400 genes, including CD2, CCR7, and CD27, were downregulated, indicating impaired T cell activity and compromised immune memory formation.

Additionally, trogocytosis facilitates bidirectional transfer of membrane proteins between CAR-T and tumor cells. Tumor cells reduce antigen density via trogocytosis to evade CAR-T killing. As shown in **Figure 2**, CAR-T cytotoxicity was significantly weaker against BALL-1 with low CD20 expression compared to high-expressing cells. Moreover, CD20-negative K562 cells failed to induce immune memory in CAR-T cells, indicating stable, high-level tumor antigen expression is essential for sustained CAR-T activity. Together, trogocytosis challenges CAR-T therapy by causing antigen loss and CAR-T fratricide. Effectively regulating this process is crucial for enhancing antitumor efficacy.

Bryostatin, a macrolide from Bugula neritina, modulates PKC activity, an effector in GPCR systems that influences MAPK signaling and cellular responses (31). Biosynthetic analogues also show anti-tumor efficacy (49). It has reported that Bryostatin activates PKC to promote synapse formation, inhibit Aβ deposition, and improve cognition in Alzheimer’s models (30, 50, 51). Recent *in vitro* studies indicated it enhances CD22 CAR-T and CAR-NK antitumor activity by upregulating CD22 on tumor cells and via antigen-nonspecific mechanisms (31).

In this study, we found that Bryostatin significantly increased CD20 expression in tumor cells and upregulated CD20 mRNA and protein in ALL-derived PBMCs at 1 ng/ml, without markedly affecting tumor cell viability. Mechanistically, Bryostatin promotes MEK/ERK-mediated phosphorylation of CREB (e.g., Ser133), enhancing its binding to the cAMP response element in the CD20 promoter (52). Furthermore, Bryostatin enhanced the cytotoxicity of CD20 CAR-T against Raji and BALL-1 cells, promoted memory phenotype formation, sustained CAR-T persistence, and stimulated CAR-T proliferation without inducing cytotoxicity. Interestingly, a recent study indicated that Bryostatin can enhance the proliferation of exhausted CD8^+^ T cells by upregulating MAPK11 expression (53). As both MAPK11 (p38) and ERK are members of the MAPK pathway and are regulated by upstream MEK kinases, this finding further supports the role of Bryostatin in functionally reinvigorating exhausted T cells. Consistent with *in vitro* findings, Bryostatin also enhanced the efficacy of CD20 CAR-T therapy in Raji lymphoma-bearing mice. This improvement was associated with increased CD20 expression in tumor tissues and enhanced infiltration of CAR-T cells (**Figure 6**).

The Bryostatin concentrations employed in this study are clinically relevant, pharmacologically achievable, and align with established safe dosing regimens, thereby supporting the translational potential of our findings. The 1 ng/mL Bryostatin concentration used *in vitro* corresponds to levels known to upregulate target antigens on B-cell tumors without PKC depletion (31). This concentration is clinically attainable: in phaseIIa trials, 25μg/m² infusion achieved ng/mL plasma levels and PKC activation within 1–2h (54, 59). Our *in vivo* dose (40μg/kg) aligns with established preclinical CAR-T-enhancement studies (32, 55). Clinically, 20-40μg/m² (∼34-72μg total per infusion) has proven safe and effective in phaseI/II trials (56–58). Bryostatin exhibits a favorable safety profile in >1,400 patients, with dose-limiting toxicity (mainly myalgia) typically occurring at >50μg/m², above doses needed for PKC activation (27, 58). Thus, the MEK/ERK-dependent synergy identified here can be feasibly implemented at tolerated doses, offering a translatable strategy to counter antigen loss in CAR-T therapy.

Therefore, by enhancing target antigen density, countering antigen loss, and promoting T-cell infiltration, all within a clinically safe dosing framework, Bryostatin represents a promising combinatorial strategy to overcome major limitations of CAR-T therapy. However, certain limitations remain. Although Bryostatin can upregulate tumor antigens through ERK signaling activation, its potential impact on CAR-T cell activity, particularly whether it may restrain trogocytosis during immune responses, requires further exploration in subsequent studies.

## Data Availability

The original contributions presented in the study are included in the article/**Supplementary Material**. Further inquiries can be directed to the corresponding author/s.

## Supplementary material

Figure Abstract Bryostatin binds to MEK and activates it, and then induces CD20 transcription through the cascade activation of the ERK/CREB signaling pathway.

Supplementary Figure 1 The structure feature of CAR construct and preparation of CAR-T cells. **(A)** The sequence of CD19 or CD20 chimeric antigen receptors is determined byCD19 or CD20 scFv, CD28-hing, 4-1BB co-stimulatory region, and CD3ζ activation domain. **(B)** Percentage of CAR+ T cells. 4 days following T cell transduction, T cells were stained with biotinylated protein L (Thermo Scientific) followed by PE-streptavidin (BD). Percentage of CAR+ T cells were detected by Flow cytometry.

Supplementary Figure 2 Percentage of CD20 on Raji cell surface co-cultured with anti-CD20 CAR T cells for 3 or 12h (n = 3 independent experiments). *P<0.05, **P<0.01.

Supplementary Figure 3 Percentage of CD19 on BALL-1 cell surface co-cultured with anti-CD19 CAR T cells for 3 (n = 3 independent experiments). *P<0.05, **P<0.01.

Supplementary Figure 4 Chord plot of GO enrichment analysis. Genes are ranked based on log2FC value (high to low). Each chord connects the gene with its associated pathway. **(A, B)** are genes with high or low expression respectively in trog-positive CD20 CAR-T.
