# Supplementary figures and images for: Bryostatin enhances CD20 CAR-T therapy efficacy against B-cell lymphoma by overcoming trogocytosis-mediated antigen loss
Source: Front Immunol. 2026 Jan 22;16:1748634. doi: 10.3389/fimmu.2025.1748634 (PMC12873546; doi:10.3389/fimmu.2025.1748634)

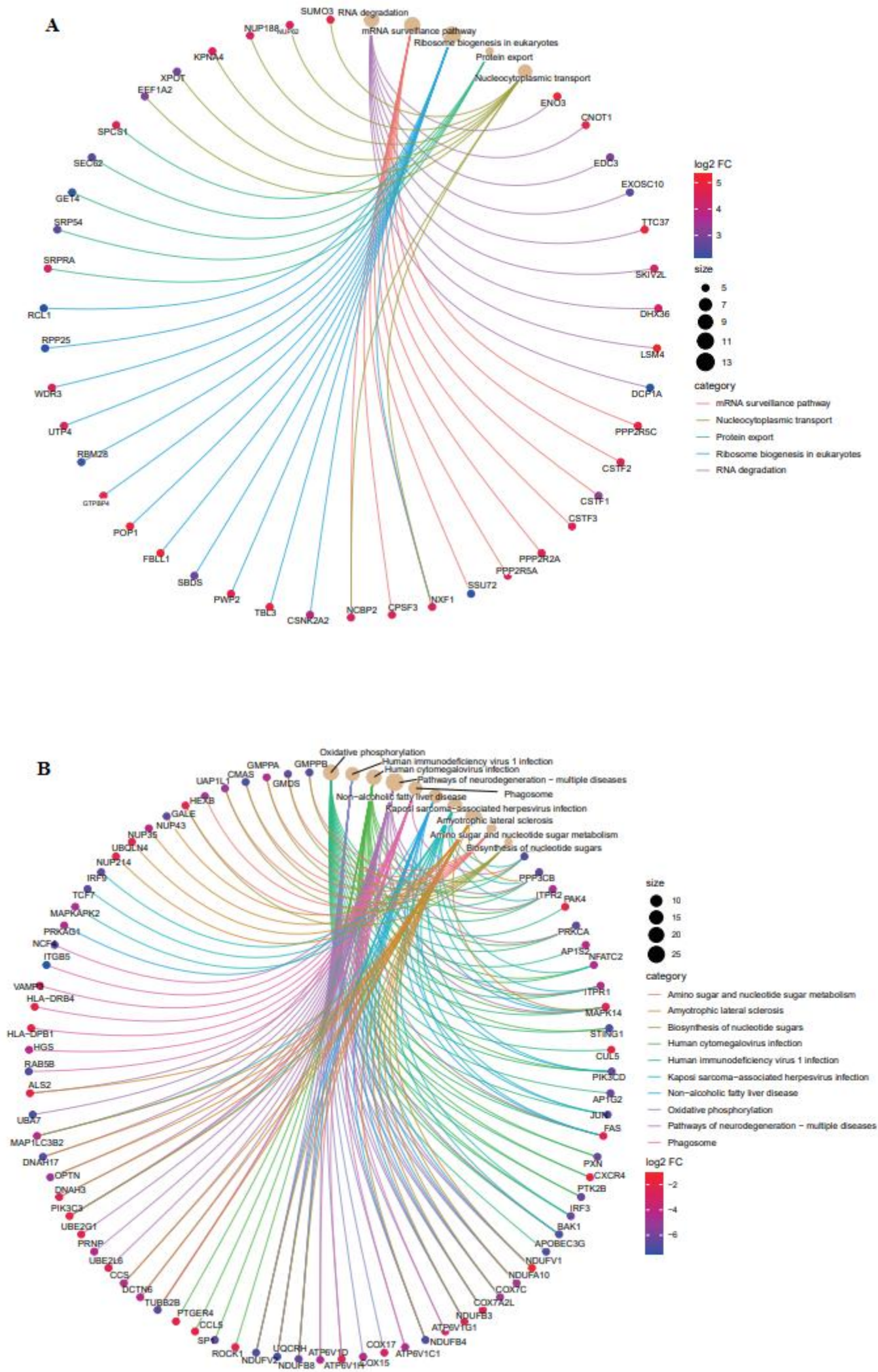

Fig.S4

Supplement: Supplementary file 1 [file DataSheet5.pdf]

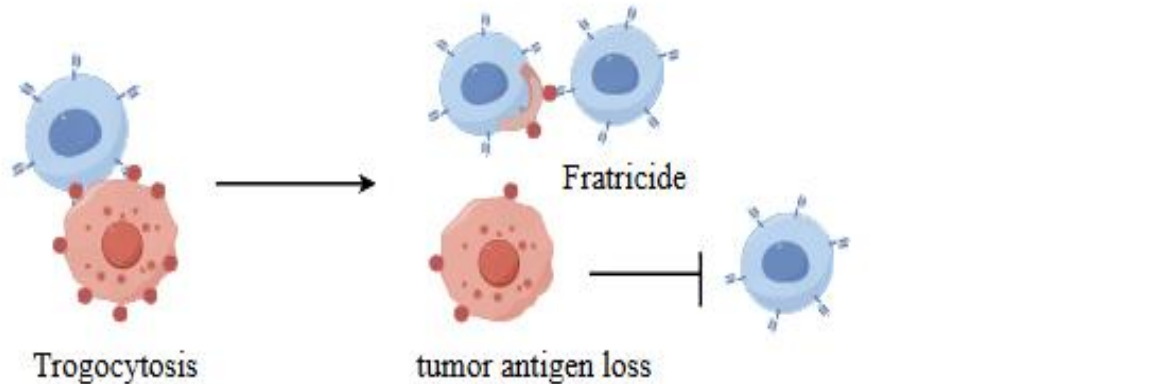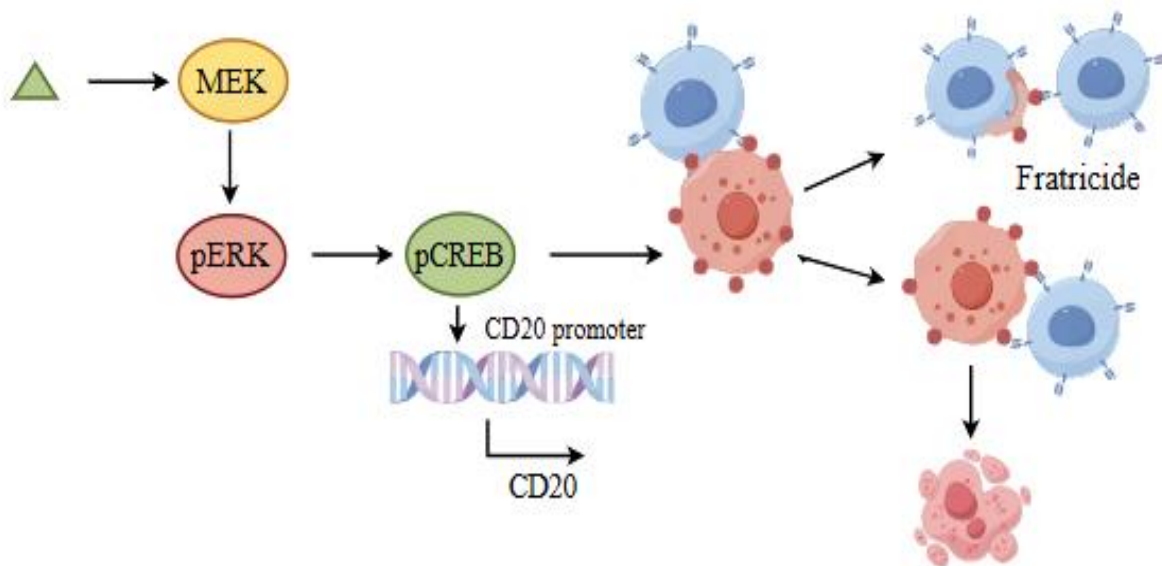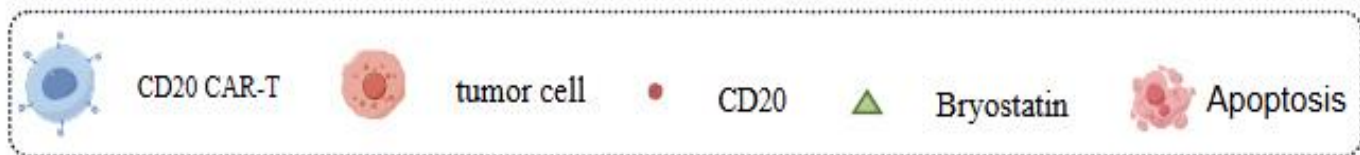

Fig.Abstact

Supplement: Supplementary Figure 1 — The structure feature of CAR construct and preparation of CAR-T cells. (A) The sequence of CD19 or CD20 chimeric antigen receptors is determined byCD19 or CD20 scFv, CD28-hing, 4-1BB co-stimulatory region, and CD3ζ activation domain. (B) Percentage of CAR+ T cells. 4 days following T cell transduction, T cells were stained with biotinylated protein L (Thermo Scientific) followed by PE-streptavidin (BD). Percentage of CAR+ T cells were detected by Flow cytometry. [file DataSheet1.pdf]

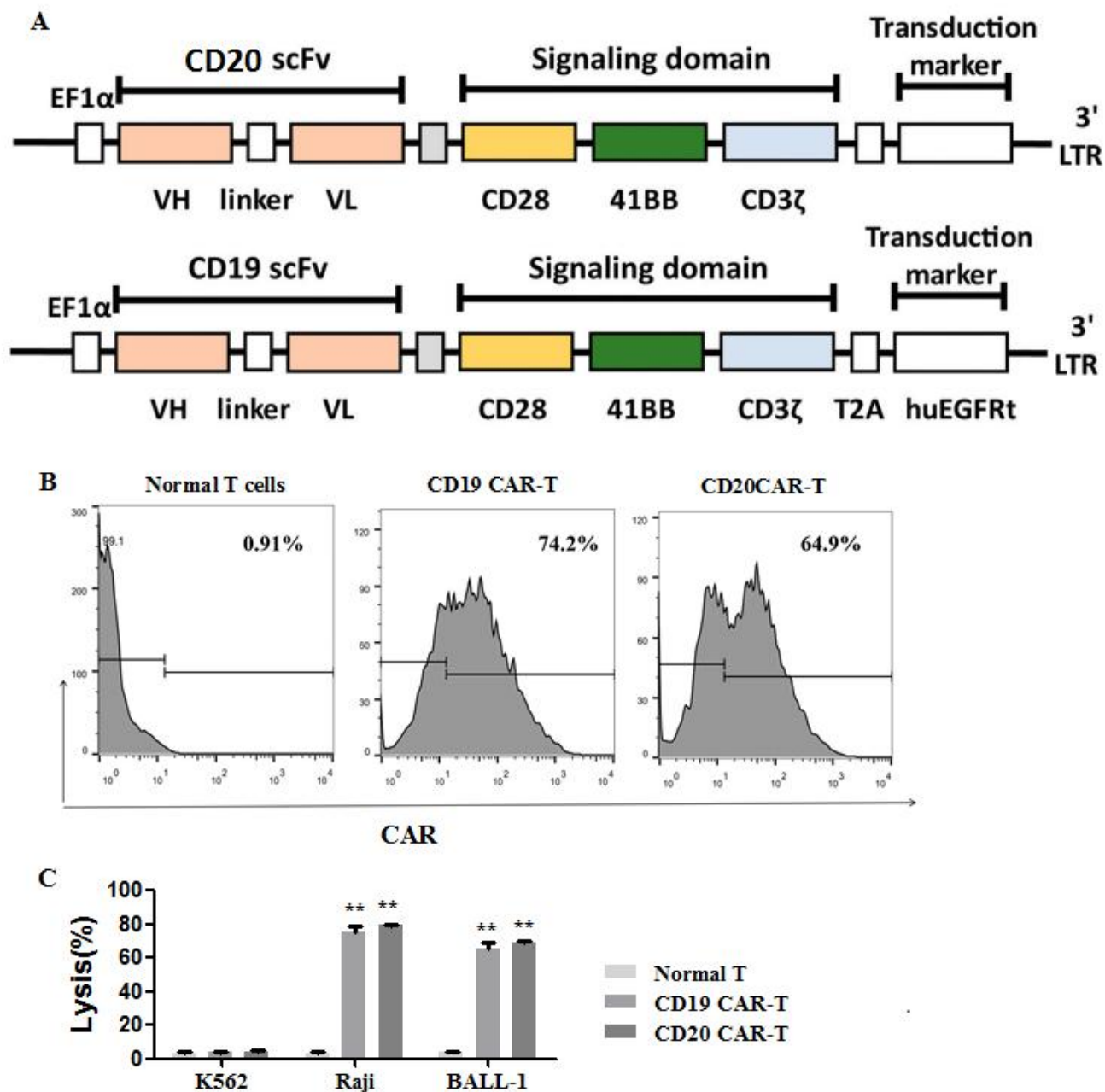

Fig.S1

Supplement: Supplementary Figure 2 — Percentage of CD20 on Raji cell surface co-cultured with anti-CD20 CAR T cells for 3 or 12h (n = 3 independent experiments). *P<0.05, **P<0.01. [file DataSheet2.pdf]

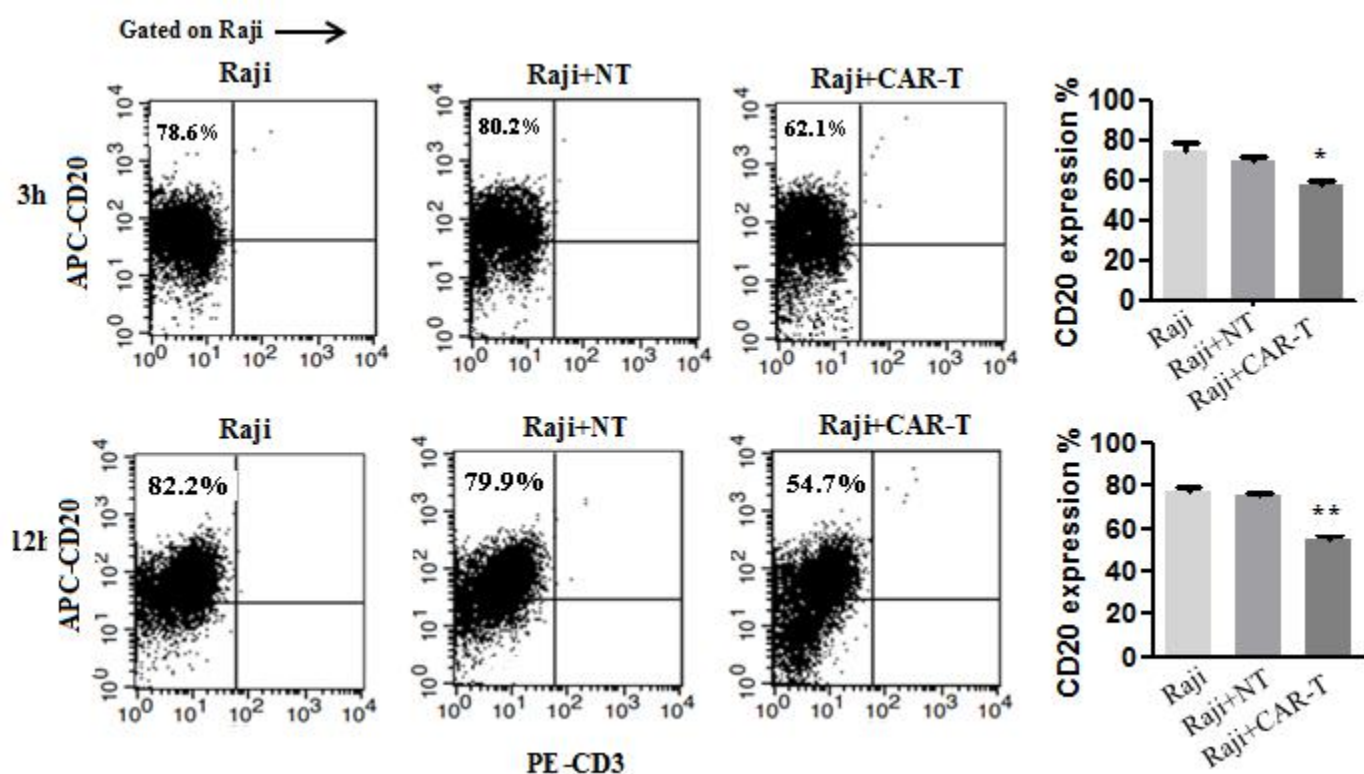

Fig.S2

Supplement: Supplementary Figure 3 — Percentage of CD19 on BALL-1 cell surface co-cultured with anti-CD19 CAR T cells for 3 (n = 3 independent experiments). *P<0.05, **P<0.01. [file DataSheet3.pdf]

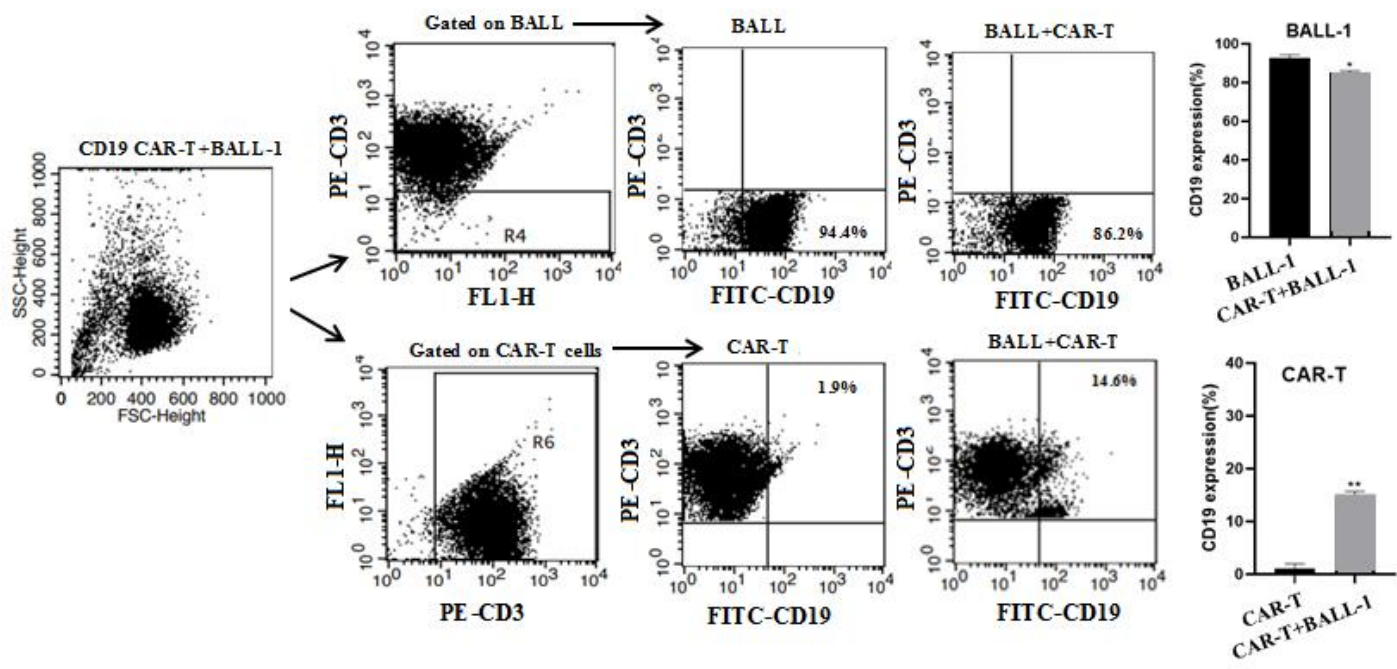

Fig.S3

Supplement: Supplementary Figure 4 — Chord plot of GO enrichment analysis. Genes are ranked based on log2FC value (high to low). Each chord connects the gene with its associated pathway. (A, B) are genes with high or low expression respectively in trog-positive CD20 CAR-T. [file DataSheet4.pdf]
